# Attack Robustness and Centrality of Complex Networks

**DOI:** 10.1371/journal.pone.0059613

**Published:** 2013-04-02

**Authors:** Swami Iyer, Timothy Killingback, Bala Sundaram, Zhen Wang

**Affiliations:** 1 Computer Science Department, University of Massachusetts, Boston, Massachusetts, United States of America; 2 Mathematics Department, University of Massachusetts, Boston, Massachusetts, United States of America; 3 Physics Department, University of Massachusetts, Boston, Massachusetts, United States of America; 4 Physics Department, University of Massachusetts, Boston, Massachusetts, United States of America; Wake Forest School of Medicine, United States of America

## Abstract

Many complex systems can be described by networks, in which the constituent components are represented by vertices and the connections between the components are represented by edges between the corresponding vertices. A fundamental issue concerning complex networked systems is the robustness of the overall system to the failure of its constituent parts. Since the degree to which a networked system continues to function, as its component parts are degraded, typically depends on the integrity of the underlying network, the question of system robustness can be addressed by analyzing how the network structure changes as vertices are removed. Previous work has considered how the structure of complex networks change as vertices are removed uniformly at random, in decreasing order of their degree, or in decreasing order of their betweenness centrality. Here we extend these studies by investigating the effect on network structure of targeting vertices for removal based on a wider range of non-local measures of potential importance than simply degree or betweenness. We consider the effect of such targeted vertex removal on model networks with different degree distributions, clustering coefficients and assortativity coefficients, and for a variety of empirical networks.

## Introduction

Many complex interacting systems can be naturally represented as networks, where the components of the system are represented by the vertices of the network and the interactions between the components are represented by edges connecting vertices in the network [1], [2]. In the last decade the study of networks has become an important area of research in many disciplines, including physics, mathematics, biology, computer science, and the social sciences [3]–[5]. There are numerous notable examples of networks in many fields of study (for more detailed discussions of many of these examples see, for instance, [6]). Important examples of technological networks are the Internet (in which the vertices are computers and associated equipment and the edges are the data connections between them) and the World Wide Web (in which the vertices are web pages and the edges are hyperlinks). Examples of important biological networks include: metabolic networks (in which the vertices represent metabolites and the edges connect any two metabolites that take part in the same reaction), protein-protein interaction networks (where vertices represent proteins and two proteins that interact biologically are connected by an edge), food webs (in which species are represented by vertices and edges represent predator-prey relationships between the species), and neural networks (where the vertices represent neurons and the edges represent neural connections). Social networks (in which the vertices represent individuals or groups and the edges represent some type of connection between them, such as acquaintance between individuals) also provide many interesting and important examples of networks.

The fundamental objective in studying the behavior of networks is to obtain insight into the complex systems they represent. An important aspect of this is to understand the effect of failure of the individual components on the performance of the whole networked system [7]. The detailed motivation for studying this effect depends on the particular networked system under consideration. For instance, it is clearly important to know how the failure of individual routers in the Internet affects the overall function of the network. Similarly, if the network in question is a contact network on which a disease can spread, then it is critical to understand how the effective removal of vertices from the network (e.g. through vaccination) affects the spread of the disease. It is clear from examples such as these that identifying those vertices that most crucially affect the function of a networked system is often of great importance. In some cases (such as the Internet) we wish to identify these vertices so that the most crucial elements of the system can be protected from failure or attack, and the functioning of the whole system can be effectively maintained. In other cases the goal is to identify the key vertices in a network so that the whole system can be most effectively degraded by their removal. Situations in which this latter goal pertains include contact networks for infectious diseases, and criminal and terrorist networks.

The precise degree to which a complex networked system continues to function as the individual components which constitute it are degraded will typically depend on subtle features of the dynamics of the system. At a somewhat cruder level, however, we may ignore the details of the particular dynamical system defined on the network and focus instead on how the *structure* of the network changes as it is degraded through the removal of vertices. This approach is justified because it is usually reasonable to assume that if a network has been so degraded by the removal of vertices that the largest connected part of the network is sufficiently small (say, only 10% of the size of the original network) then any sensible dynamical process will be unable to function on the degraded network in an effective way.

A considerable amount of effort has been devoted to understanding how network structure changes when vertices are removed and we will briefly review the existing literature on this subject. By far the largest amount of work on the robustness of networks has focused on the effect of removing vertices uniformly at random or in decreasing order of their degree. References [7]–[10] study this question in considerable detail, and also discuss the related issue of percolation on networks. Much of this work is reviewed in [6]. In related work, [11] studies the efficiency of networks under the removal of vertices uniformly at random or in decreasing order of their degree. Interesting recent work has considered the evolution of network topologies that are robust to the removal of vertices based on their degree [12], [13]. Much less, however, is known about how the structure of networks change when vertices are removed according to more subtle non-local measures of their possible importance. The most significant previous study of this latter question is [14], in which the effect of removing vertices both in decreasing order of degree and of betweenness centrality is considered. Related work considering the effect of removing vertices based on betweenness is also described in [15]. In addition to these references there are more distantly related works that study the effect of removing vertices based on various centrality measures on certain processes defined on networks. Reference [16] studies the effect of removing species in a food web according to eigenvector centrality on coextinctions of other species in the food web. The effect of vaccinating individuals in a contact network according to different centrality measures on the spread of an epidemic is discussed in [17].

The purpose of this paper is to extend these investigations by systematically studying the effect on network structure of removing vertices according to a wider variety of non-local schemes. We investigate the effect of removal schemes based on degree, betweenness, closeness, and eigenvector centrality on a wide variety of model networks, including those with power-law and exponential degree distributions, different clustering coefficients, and different degrees of assortativity. In addition, we study, the consequences of these methods of vertex removal for a variety of empirical networks, such as, neural networks, protein-protein interaction networks, and social networks. In all cases we quantify the vulnerability of a network to a given scheme of vertex removal by a single numerical quantity, which allows a precise comparison of the efficacy of different removal schemes to be made.

## Analysis

### Percolation and Robustness

Understanding the robustness of networked systems against the failure of their component parts is closely related to the study of percolation on networks. The process that results from taking a network and removing some fraction of its vertices (together with the edges connected to the vertices) is refered to as *percolation*. Percolation provides a natural model for studying the robustness of networked systems [8], [9]. For example, the failure of routers in the Internet, or the vaccination of individuals to prevent the spread of a disease, can be represented formally by the removal of the corresponding vertices from the relevant networks. Although a router that has failed or an individual that has been vaccinated is still present in the network, from a functional point of view it may as well have been removed.

One of the key aspects of studying percolation on a network 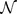 is to understand how the size of the largest component changes as vertices are removed from the network [8], [9]. This is clearly relevant to the issue of network robustness since if the size of the largest component is sufficiently small, relative to the original size of the network, it is reasonable to assume that the networked system will be unable to function in any sensible way. For an initial network 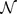 of size 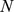, let 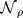 be the network that results from removing a fraction 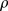 of the vertices according to some specified procedure. We will denote by 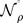 the largest component of 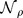. The key quantity that we will study here is the size 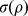 of 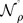 relative to the initial size of the network 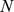: that is, 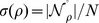, where 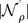 denotes the number of vertices in 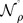. Computing 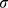 as a function of 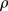 allows us to quantify how the robustness of a network depends on the fraction of vertices that are removed.

There are many ways in which vertices can be removed from a network. The simplest is to remove the vertices uniformly at random from the network. Studying how 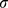 depends on 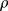 when vertices are removed uniformly at random is closely related to the classical percolation process (in which vertices are removed at random from a low dimensional lattice, such as the two-dimensional square lattice, see [18]). There are other ways in which vertices can be removed apart from uniformly randomly and here we will follow [8], and also [6], and use the term percolation to cover any specific procedure for vertex removal. One particularly natural procedure is to remove vertices in order of their degrees, from highest to lowest [7]–[10]. More generally, we can consider removing vertices according to any quantity which aims to measure the importance of different vertices. The concept of a centrality measure attempts to provide precisely such a quantification of the importance of the vertices in a network. The simplest centrality measure is just the degree of the vertex. Other well-known centrality measures attempt to quantify the significance of a vertex by counting how many short paths between other pairs of vertices pass through the vertex in question or by calculating how close on average a given vertex is to all other vertices in the network. The centrality measures of relevance to us here are discussed in more detail in the next section.

Once a suitable centrality measure has been fixed, we can compute 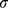 as a function of 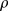 for removing vertices in decreasing order of that centrality measure. The robustness of a network under this type of vertex removal can be quantified by the 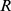
- *index*, which is defined by [13]

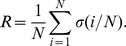



The normalization factor 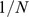 allows the robustness of networks of different sizes to be compared [13]. It is straightforward to show that for any scheme of removing vertices from any network, 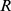 attains its minimum value of 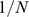 on the star graph and its maximum value of 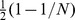 on the complete graph. Thus, for any network and method of vertex removal, 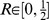. Consequently, we define the 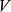
- *index* 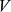, which measures the *vulnerability* of a network to a given scheme of vertex removal, to be the complementary quantity to 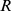,
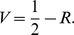



For uniform random removal of vertices and for removal of vertices in decreasing order of degree the percolation process on networks has been carefully studied, and elegant analytical results have been obtained in the limit of large network size (i.e. in the limit 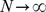) [8], [9]. One of the conclusions of these studies is that scale-free networks are very robust to uniform random removal of vertices, but highly susceptible to removal which targets the highest degree vertices. These results are often paraphrased by saying that scale-free networks are robust to “error”, but vulnerable to “attack”.

Here we study the effect on network robustness of targeted removal of vertices according to a number of more complex centrality measures than simply degree. Since these centrality measures are subtle non-local measures of a vertex’s significance it seems unrealistic to anticipate any all-embracing analytical theory of the corresponding percolation process, and hence our present work is computational in nature.

### Centrality Measures

The concept of a *centrality measure* attempts to identify which vertices in a network are the most important or central [19], [20]. A number of different measures of centrality have been proposed for networks, and here we will focus on the four most common: degree centrality, eigenvector centrality, closeness centrality, and betweenness centrality.

The networks that we consider here will be assumed to be simple (i.e. no multi-edges or self-edges) and undirected. The number of vertices in the network will be denoted by 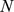 and the number of edges by 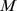. Thus, for any such network 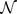, the adjacency matrix 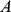, defined by
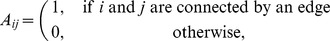
is real symmetric, and consequently has real eigenvalues. Since 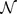 is assumed to be simple it follows that 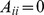. We also introduce the notation 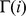 for the set of neighbors of vertex 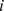: 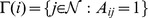. We now recall the definitions of the centrality measures that will be important in this paper – see [6] for a more detailed discussion.

#### Degree Centrality

The simplest measure of the centrality of a vertex in a network is just the degree of the vertex. When degree is used as a centrality measure it is often referred to as *degree centrality*. It is clear from considering various examples of networks, such as social networks or citation networks, that the number of edges a given vertex is connected to (i.e. the vertex’s degree centrality) may often be a good measure of the vertex’s importance. Thus, if **A** is the adjacency matrix of the network 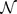 then the degree centrality of a vertex 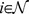 is simply the degree 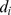 of 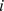 given by
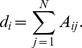



#### Eigenvector Centrality

Another widely employed centrality measure, which can be viewed in a sense as a refinement of degree centrality, is *eigenvector centrality* [21]. Whereas degree centrality ranks a vertex as being important if it is connected to many other vertices, eigenvector centrality is based on the more subtle notion that a vertex should be viewed as important if it is linked to other vertices which are themselves important. This notion naturally leads to a recursive definition of eigenvector centrality [22]: the eigenvector centrality 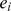 of a vertex 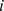 is defined to be proportional to the sum of the eigenvector centralities of the vertices it is connected to, i.e.
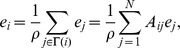
where 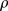 is a constant. It will also be assumed that the eigenvector centrality of each vertex is non-negative: i.e. 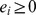, for all 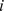. If 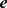 is the vector of eigenvector centralities with elements 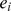, then we can write the last equation in matrix form as 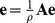, or



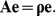



It follows from the non-negativity of ***e***, using the Perron-Frobenius theorem [23], that the eigenvector centralities of the vertices in the network are given by the elements of the eigenvector of ***A*** corresponding to the dominant eigenvalue. The eigenvector centrality of a vertex has the attractive feature that it can take a large value either by the vertex being connected to many other vertices or by it being connected to a small number of important vertices.

The eigenvector centrality also has an interesting relation to a simple dynamical process on the network [6]. To see this let 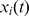 be a real valued dynamical variable associated to vertex 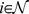, at time 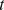. We can define a discrete dynamical process on 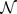 by
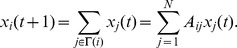



This can be written in vector form as 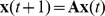, where 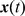 is the vector with elements 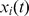. Given an initial vector 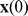, the vector 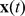, at time 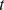, is
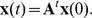



We can obtain the asymptotic behavior of this system by writing 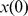 as a linear combination of the eigenvectors 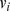 of ***A***:
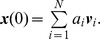



Thus,
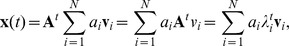
where 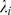 is the eigenvalue of ***A*** corresponding to eigenvector 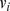. Let 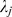 be the largest eigenvalue and denote it by 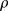. Then



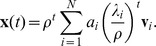



Since, 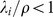, for all 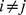 we have that 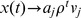 as 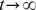. Therefore, in the asymptotic limit the value of the dynamical variable associated to a vertex 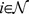 is simply proportional to 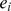, the eigenvector centrality of 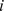. Thus, the eigenvector centrality can also be interpreted as a measure of the relative asymptotic distribution of the dynamical variable 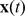 over the vertices of the network.

The *power method*, which is an application of the equation 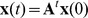, provides an efficient method for computing the eigenvalue centralities of the vertices of a network (see [6]).

#### Closeness Centrality

Closeness centrality provides a rather different measure of centrality than degree or eigenvector centrality, as it is based on the mean distance between a given vertex and all other vertices in the network [24], [25]. In order to define closeness centrality we need the notion of a *geodesic path* in a network. A geodesic path between two vertices 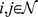 is simply a path between 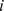 and 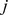 such that no path of shorter length exists (where the length of a path between 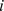 and 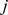 is defined to be the number of edges traversed in going from 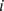 to 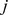). We note that geodesic paths are not in general unique, since there can be several paths between two given vertices with the same shortest length. However, at least one geodesic path always exists between any two vertices in the same connected component of a network.

Let 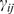 be the length of a geodesic path from 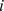 to 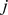 in 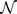. The mean geodesic distance between 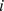 and all other vertices in the network is
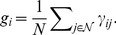
(1)


We note that it is natural to exclude the term 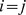 in calculating this sum, since we are calculating the mean geodesic distance between 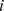 and the other vertices in the network. However, since 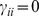 this term does not contribute to the sum, and (1) provides a convenient definition of the mean geodesic distance. We now define the *closeness centrality* 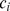 of a vertex 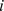 by
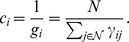



This quantity takes high values for vertices that are only a short geodesic distance from many other vertices in the network, and is a natural measure of centrality which is widely used in network studies.

Here we adopt the standard convention that if a network has more than one component then the closeness centrality of a vertex 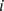 is calculated as the reciprocal mean geodesic distance from the vertex to all other vertices in the same component – that is, the sum in (1) is taken over only those vertices in the same component as 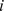 (see, for example, [6]). We also note that there is an efficient algorithm for computing closeness centrality [26].

#### Betweenness Centrality

A still different notion of centrality is provided by *betweenness centrality*, which measures how many short paths between vertices in the network pass through a given vertex [27]. To be more precise let us first consider a network  for which there is a unique geodesic path between any two vertices (see [6]). If we consider the set of geodesic paths between all pairs of vertices 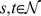, then the betweenness centrality of a vertex 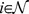 is defined to be the number of these paths that pass through 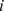. Thus, if we define
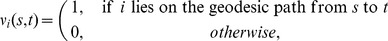
then the betweenness centrality 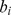 of 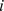 is given by



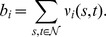



Let us now consider the general case in which the network may have more than one geodesic path connecting a pair of vertices (see [6]). The standard way of extending the notion of betweenness centrality to this situation is to give each geodesic path between two vertices 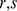 a weight equal to the reciprocal of the number of geodesic paths from 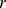 to 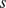, and then to define the betweenness centrality of a vertex to be the sum of the weights of all geodesic paths that pass through it.

The betweenness centrality in the general case can, therefore, be expressed in terms of the number of geodesic paths from 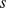 to 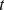 that pass through 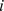, 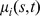, and the total number of geodesic paths from 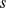 to 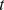, 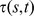, as:
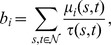
where we define 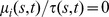 if both 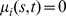 and 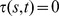.

Betweenness centrality is interestingly different from the preceding three measures of centrality in that a vertex may have a high betweenness centrality while being connected to only a small number of other vertices, which need not have great importance themselves. This is because vertices that act as “bridges” between groups of other vertices will typically have high betweenness centrality. Thus, vertices with high betweenness centrality play an important role in governing the flow of information through a network. This feature of betweenness centrality makes it an important centrality measure for a wide range of social, technological, and biological networks. Here again there is an efficient algorithm to calculate betweenness centrality [28].

## Results

In this section we investigate the effect on network structure of removing vertices according to some specified procedure. Here the procedure of interest will be to remove vertices in order of their importance, as determined by one of the four centrality measures discussed in the previous section. That is, for any network under consideration, we determine the importance of the vertices in the network by calculating the degree, betweenness, closeness, or eigenvector centralities of the vertices, and then compute the effect on the size of the largest connected component of the network of removing a given fraction of the vertices in decreasing rank order with respect to the specified centrality measure.

It is important to note that there are two distinct schemes according to which a given centrality measure can be used to target the removal of vertices in a network. In the first, the centrality measure is calculated for all vertices in the network, and then a specified fraction of the vertices are removed in order of the centrality measure, from highest to lowest. We shall refer to this procedure as *simultaneous targeted attack*. Simultaneous targeted attack is a natural scheme for removing vertices in various situations. For example, in the context of vaccinating the individuals in a population to prevent the spread of an infectious disease, it is reasonable to compute some measure of the significance of each vertex in the contact network for acquiring and/or transmitting the disease, and then vaccinate some fraction of the population in decreasing order of that measure. When the centrality measure is simply degree this type of vaccination scheme has been well-studied [10].

In the second scheme, the centrality measure is calculated for all vertices in the initial network, and the vertex with highest centrality measure is removed. The removal of this vertex results in a new network in which the centrality measures of the remaining vertices may be different from the values that were calculated for them previously. We, therefore, recalculate the centrality measures of all vertices in the new network and again remove the highest ranked. This process of recalculation of centrality measures and removal of the highest ranked vertex is continued until the desired fraction of vertices has been removed. We shall refer to the latter procedure as *sequential targeted attack*.

Sequential targeted attack is the more natural method of vertex removal in certain situations. One example of such a situation is the identification of the most vulnerable vertices in the Internet in order to protect the network’s function. Since the failure of different routers can realistically be assumed to be distributed over some period of time, and since the failure of any one router will affect the importance of the remaining ones, it is appropriate to model the vulnerability of the system by sequential targeted attack.

A second example of the appropriateness of sequential targeted attack arises in analyzing the effect of vertex removal in biological networks, such as protein-protein interaction networks. If a mutation in the gene coding for a particular protein results in the protein being biologically inactive (e.g. being unable to form a protein interaction complex) then the corresponding vertex in the protein-protein interaction network is effectively removed. If the mutation is not lethal then a subsequent gene mutation could occur in a later generation resulting in the removal of another vertex in the protein-protein interaction network of the organism. This process could, in principle, continue for a number of mutations. In such a situation vertex removal occurs sequentially and the vulnerability of the protein-protein interaction network should be modeled by sequential targeted attack.

Here we study the percolation processes on complex networks for both simultaneous targeted attack and sequential targeted attack based on degree, betweenness, closeness, and eigenvector centrality. The effect of simultaneous and sequential targeted attack on certain networks according to only degree and betweenness was discussed in [14], and also in [15].

### Simultaneous Targeted Attack

We now study the robustness of a variety of model and real-world networks to simultaneous targeted attack according to degree, betweenness, closeness, and eigenvector centrality measures. For each network we calculate each of these four centrality measures for all vertices, and then compute the fractional size of the largest component 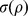, when a fraction 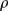 of the vertices have been removed in decreasing order of a specified centrality measure. We study this process for model networks with power-law and exponential degree distributions, for model networks with clustering and with assortativity and disassortativity, and for a number of empirical networks.

**Figure 1:**
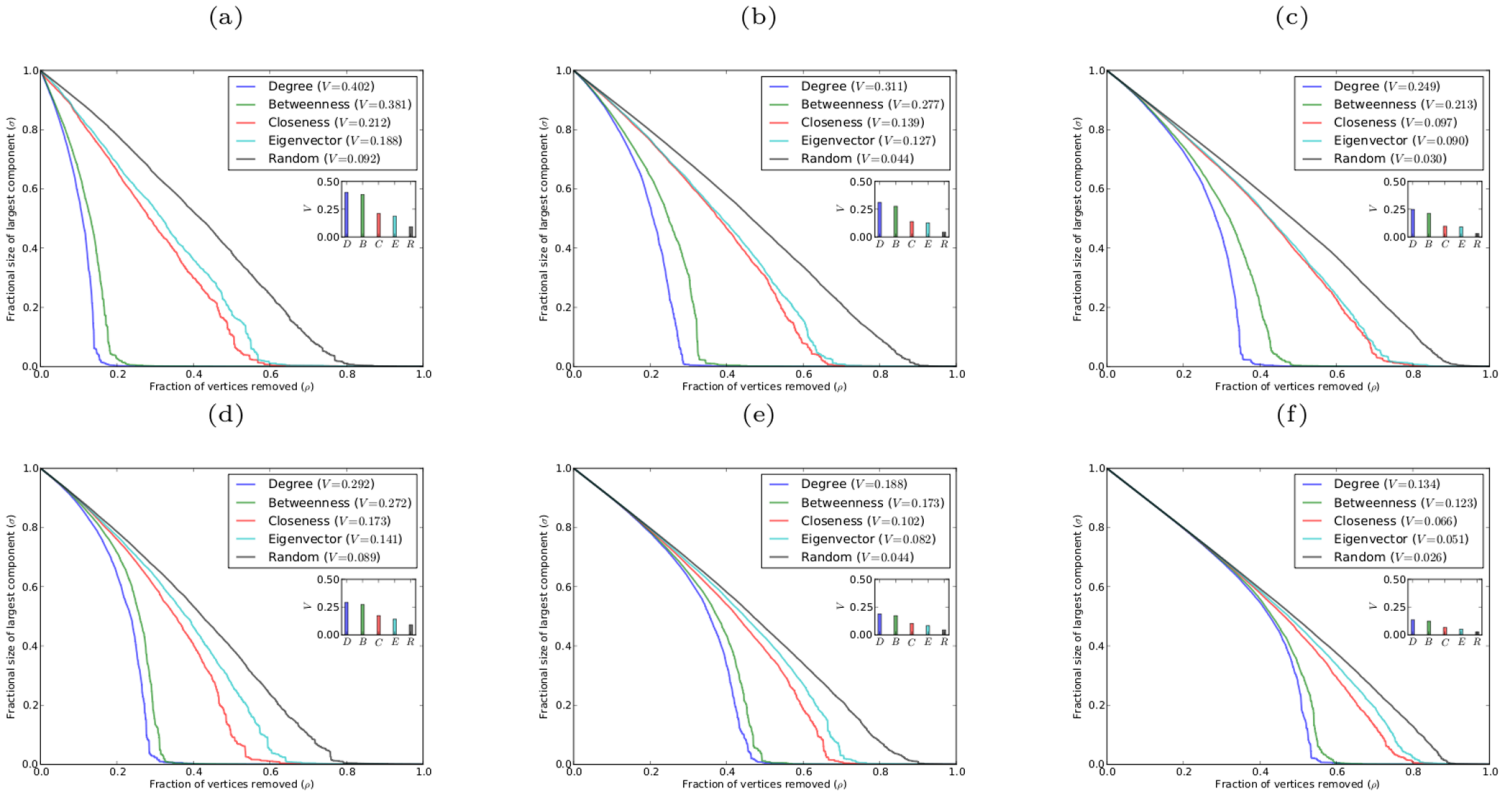
Robustness against simultaneous attack for model networks with power-law and exponential degree distributions, with 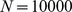, and different average degree 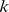. (a)(b)(c) scale-free networks with 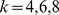, respectively; (d)(e)(f) exponential networks with 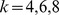, respectively.

Figure 1 shows robustness results for networks with power-law degree distribution, generated using the Barabási-Albert preferential attachment model (in which each new vertex entering the network attaches to a fixed number of existing vertices chosen in proportion to their degrees [2]), and for networks with exponential degree distribution, generated using the growing random graph model (in which each new vertex entering the network attaches to a fixed number of existing vertices chosen uniformly at random [29]). Both power-law and exponential degree distributions commonly occur in real-world networks [30].

It is apparent from both the graphs of 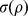 against 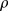 and from the corresponding 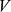-indices that networks with both power-law and exponential degree distributions are most vulnerable to simultaneous targeted attack according to degree centrality. These networks are almost as vulnerable to simultaneous targeted attack according to betweenness centrality, with attack based on closeness and eigenvector centrality being considerably less effective. Random (i.e. non-targeted) attack is much less effective at degrading the structure of these networks than targeted attack based on any of the four centrality measures. It is rather striking that degree which is a purely local centrality measure provides a more effective means of targeting vertices than any of the other centrality measures, which are non-local in nature and can account for the global structure of the network. We believe that degree centrality will prove in general to be superior to other centrality measures at exposing the vulnerability under simultaneous targeted attack of any network which lacks certain specific structural properties that would favor the efficacy of other centrality measures. For instance, the presence in a network of a large number of low degree vertices that act as “bridges” between different highly connected parts of the network might be expected to favor betweenness centrality as the most effective method of detecting highly vulnerable vertices. In the absence of any particular structural properties the best estimator of the vulnerability of a vertex under simultaneous targeted attack appears to be simply the number of neighbors that the vertex has. The networks considered in Figure 1, although having prescribed degree distributions, are essentially random in nature and thus lack any specific structural properties that would allow other centrality measures to be superior estimators of vulnerability than degree.

**Figure 2:**
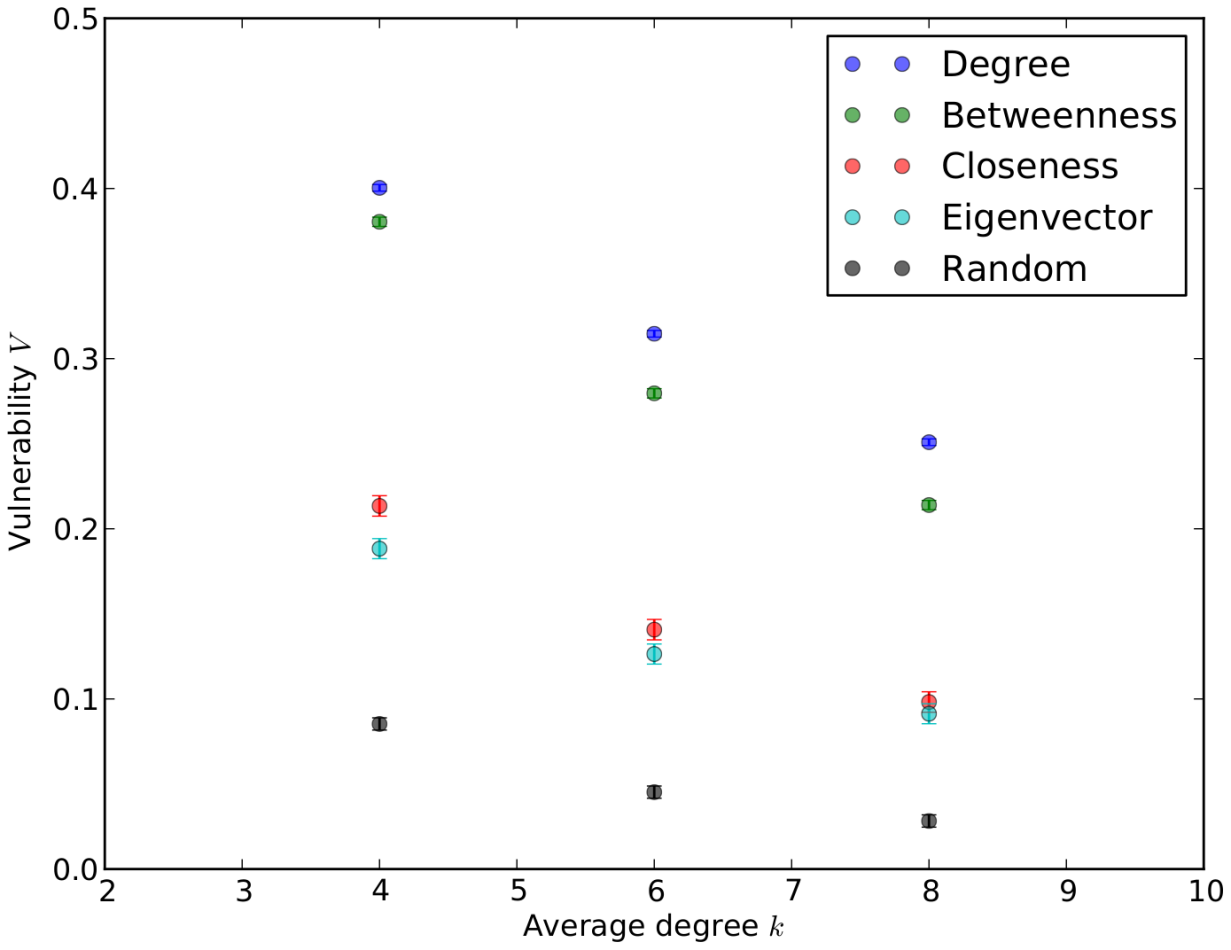
Variance in V-index for scale-free networks with 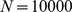 and different average degree 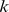. The variance is based on ten different realizations of the network for each value of 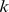, and the error bars represent the standard error.

Here we have calculated the robustness results using a single realization of each type of random network. It is important to obtain some sense of the variance in the V-index that results from different network realizations. This is shown for scale-free networks of varying mean degrees in Figure 2. We note that there is very little variance in the values of the V-index obtained from different network realizations. Thus, the robustness results obtained from a single realization of a given type of network provide a true picture of the general robustness of networks of that type.

**Figure 3:**
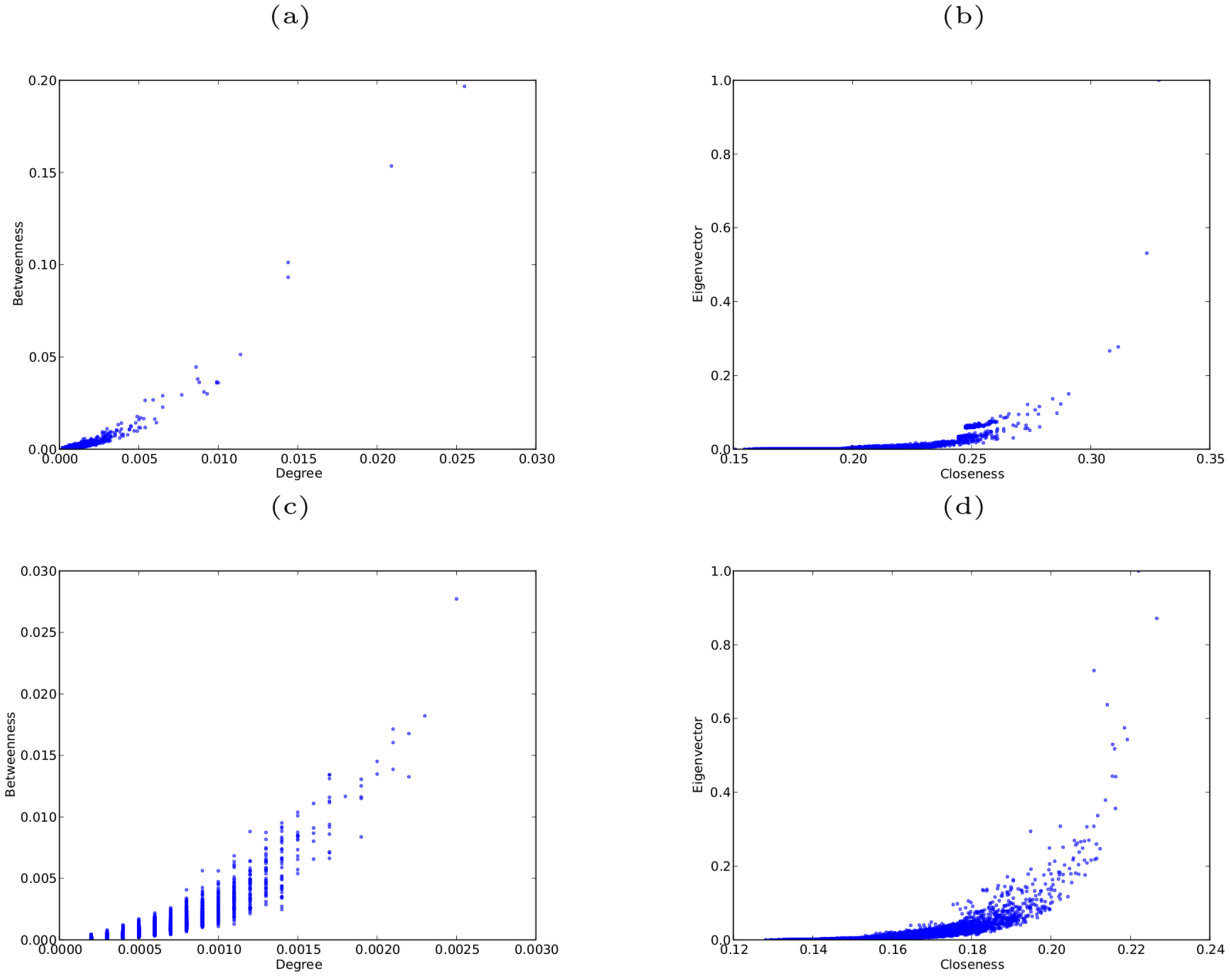
Correlations between centrality measures of power-law and exponential networks with 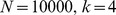. (a) degree versus betweenness, power-law network, (correlation = 0.878); (b) closeness versus eigenvector, power-law network (correlation = 0.564); (c) degree versus betweenness, exponential network (correlation = 0.843); (d) closeness versus eigenvector, exponential network (correlation = 0.608).

An interesting result, that is immediately apparent from Figure 1, is that targeting vertices according to either degree or betweenness is very similar in effect. It is clearly also the case that targeting vertices by either closeness or eigenvector centrality has much the same effect. The explanation for these similarities is that for networks with both power-law and exponential degree distributions the degree and betweenness centralities of the vertices are strongly correlated, and the closeness and eigenvector centralities are also highly correlated. These correlation results are shown in Figure 3.

A common property of real-world networks is that they have non-trivial clustering coefficient. The clustering coefficient of a network measures the average probability that two neighbors of a vertex are themselves adjacent. The local clustering coefficient 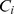 of a vertex 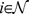 is defined to be [5].
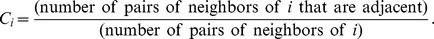



The global clustering coefficient 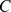 for the whole network is then defined as the mean of the local clustering coefficients 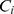 [6]:
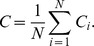



A network with 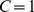 has maximal clustering, while one with 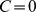 has no clustering.

**Figure 4:**
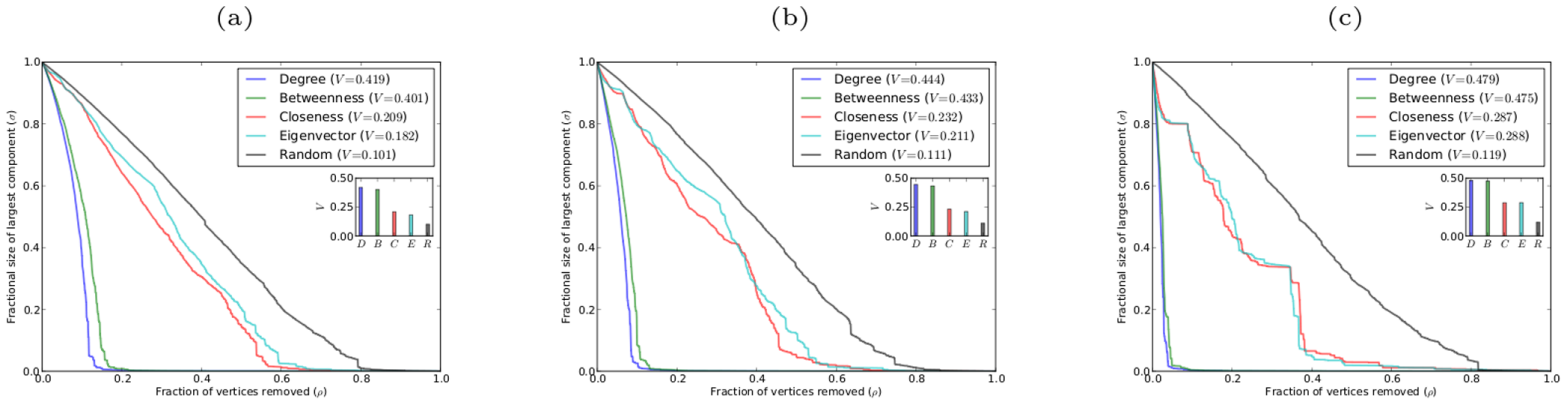
Robustness against simultaneous attack for model scale-free networks with 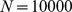, 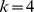, and different values of the clustering coefficient 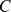. (a) 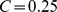; (b) 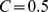; (c) 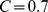.

Figure 4 shows robustness results for scale-free networks with different clustering coefficients (generated using the Holme-Kim model [31]). It is clear from the graphs of 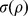 and from the corresponding 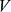-indices, that for scale-free networks with clustering, simultaneous targeted attack by degree is again most effective at exposing network vulnerability. The efficacy of simultaneous targeted attack by other centrality measures follows a similar pattern as for networks without clustering. However, as clustering increases the effectiveness of attack based on degree and on betweenness becomes almost indistinguishable, as does that of attack based on closeness and eigenvector centrality.

One additional unexpected result is that increasing clustering coefficient results in decreasing robustness to simultaneous targeted attack by any centrality measure, with the most dramatic effect being displayed for attack based on degree and betweenness. The networks with different clustering coefficients constructed using the Holme-Kim model [31] have no significant differences in their degree distributions or number of edges. There is, however, an increase in average path length with increasing clustering coefficient, which is characteristic of decreasing robustness [7]. We find that the average path lengths for the networks studied in Figure 4 are 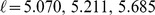 for 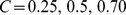, respectively. This increase in the average path length with increased clustering is consistent with the increase in the V-index with increased clustering, and supports the general conjecture that networks exhibit decreased robustness with increased clustering coefficient. This result has potentially important implications as most real-world networks have significant levels of clustering, and thus may be more fragile to targeted attack than networks with the same degree distribution but lower clustering. This result raises the possibility that one procedure for increasing the robustness of certain real-world networks (such as some technological networks) is to design them with as low a clustering coefficient as is consistent with the functional requirements of the network. Understanding the fundamental origin of the decrease in robustness of networks as their clustering coefficient increases appears to be an important topic for future research.

Another common property of real-world networks is that they possess some amount of assortativity or disassortativity [32]. Assortative networks have the property that high degree vertices tend to be connected to other high degree vertices and low degree vertices to other low degree ones. In contrast, for disassortative networks, high degree vertices tend to be connected to low degree vertices and vice versa. Social networks are usually assortative, while biological and technological networks are typically disassortative [32].

The assortativity (or disassortativity) of a network can be measured by the coefficient of assortativity 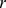, defined by [32]

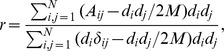
where 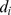 is the degree of vertex 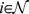 and 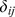 is the Kronecker delta.

Networks with 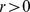 are assortative and those with 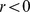 are disassortative. Networks with 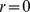 are neither assortative nor disassortative. There are a variety of algorithms for generating networks with a given degree distribution and coefficient of assortativity. Here we generate assortative and disassortative networks by applying the rewiring algorithm of [33] to a Barabási-Albert’s scale-free network. Since the rewiring procedure maintains the degree sequence of a network, this procedure results in a scale-free network with a non-zero coefficient of assortativity.

**Figure 5:**
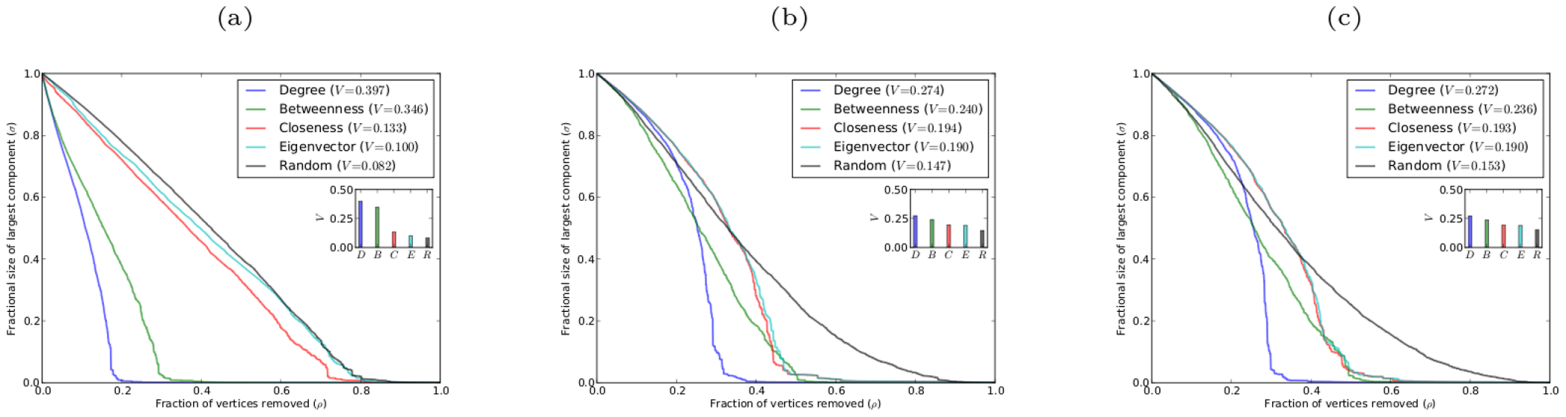
Robustness against simultaneous attack for model scale-free networks with 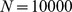, 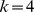, and different values of the coefficient of assortativity 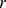. (a) 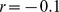; (b) 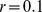; (c) 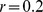.

The robustness results for such scale-free networks with different coefficients of assortativity are shown in Figure 5. These results show some interesting differences from the previous cases. For disassortative networks (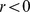), simultaneous targeted attack by degree is again the most effective means of exposing the vulnerability of a network. For such networks, the advantage of targeting vertices by degree rather than betweenness is even greater than for networks with zero degree of assortativity. This result appears to reflect the fact that for disassortative networks the high degree vertices are distributed throughout the network, and so the removal of the high degree vertices rapidly degrades the structure of the network.

In contrast, for assortative networks (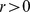), simultaneous targeted attack by betweenness is initially the most effective method of degrading the network. Once the fraction of vertices removed exceeds about 25%, targeting according to degree rather than betweenness results in the network being degraded more rapidly. This result is a consequence of the fact that for an assortative network high degree vertices are preferentially connected to other high degree vertices, and thus form a concentrated interconnected core. Consequently, the network is relatively robust against the removal of high degree vertices, since the removal of a moderate number of vertices in the core will be unlikely to dramatically affect the size of the largest connected component. In this case the non-local information concerning the global structure of the network contained in the betweenness centrality is better able to identify the most critical vertices. Since removing vertices according to betweenness centrality results in a rapid decrease in the size of the largest component in the network, it follows that once a sufficient fraction of the vertices have been removed the core of high degree vertices will have been significantly diminished. Once this point is reached targeting vertices by degree again becomes the most effective method of exposing network vulnerability. The relevant 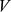-indices show that despite the differences in how assortative and disassortative networks are degraded by vertex removal according to different centrality measures, the overall effectiveness of the various attack schemes follows the same pattern as that found above for networks with zero coefficient of assortativity: namely, attack based on degree is the most effective overall and that based in eigenvector centrality is the least.

**Figure 6:**
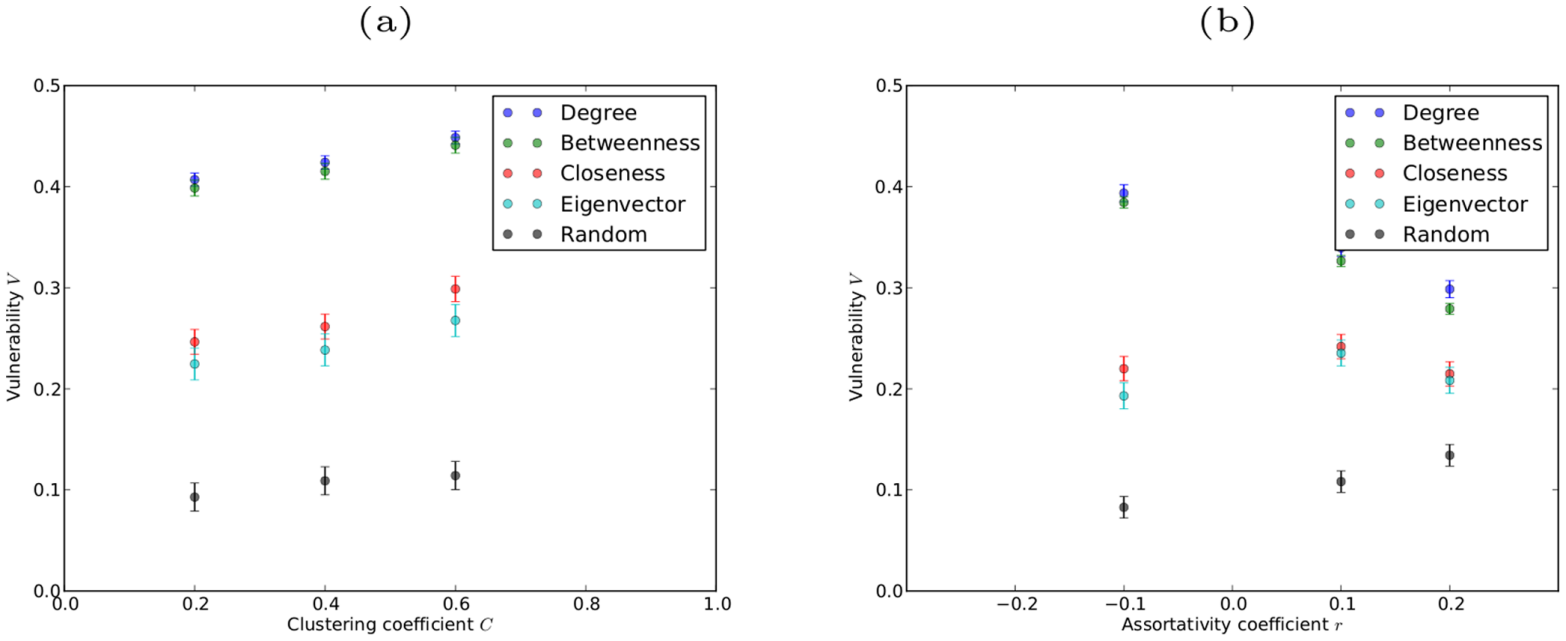
Variance in V-index for networks with 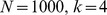 and varying clustering coefficients (a) and varying assortativity coefficients (b). The variance is based on ten different realizations of the network for each value of clustering and assortativity coefficient, and the error bars represent the standard error.

In the preceding discussion of the robustness of clustered and assortative networks we have, as before, determined the robustness results using a single realization of each type of network. Again, it is important to understand the variance in the V-index that results from different network realizations. This is shown for networks with varying clustering coefficients and assortativity coefficients in Figure 6. We note that there is little variance in the values of the V-index obtained from different network realizations, and thus, the robustness results obtained from a single realization of a given type of network gives an accurate account of the general robustness of networks of that type.

The V-indices under simultaneous targeted attack for all the networks discussed above are summarized in Table 1.

**Table 1:** The 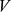-indices of model networks in the case of simultaneous attack.

	Degree	Betweenness	Closeness	Eigenvector	Random
Power-law network ( 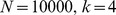 )	0.402	0.381	0.212	0.188	0.092
Power-law network ( 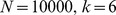 )	0.311	0.277	0.139	0.127	0.044
Power-law network ( 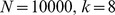 )	0.249	0.213	0.097	0.090	0.030
Exponential network (N = 10000; k = 4)	0.292	0.272	0.173	0.141	0.089
Exponential network (N = 10000; k = 6)	0.188	0.173	0.102	0.082	0.044
Exponential network (N = 10000; k = 8)	0.134	0.123	0.066	0.051	0.026
Scale-free network with clustering (N = 10000; k = 4; C = 0.25)	0.419	0.401	0.209	0.182	0.101
Scale-free network with clustering (N = 10000; k = 4; C = 0.5)	0.444	0.433	0.232	0.211	0.111
Scale-free network with clustering (N = 10000; k = 4; C = 0.7)	0.479	0.475	0.287	0.288	0.119
Scale-free network with assortativity (N = 10000; k = 4; r = −0.1)	0.397	0.346	0.133	0.100	0.082
Scale-free network with assortativity (N = 10000; k = 4; r = 0∶1)	0.274	0.240	0.194	0.190	0.147
Scale-free network with assortativity (N = 10000; k = 4; r = 0.2)	0.272	0.236	0.193	0.190	0.153

The 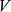-indices of model networks in the case of simultaneous attack by degree, betweenness, closeness, and eigenvector centralities.

The preceding results have focused on important types of networks that have been generated using network models. We have also studied the robustness of a number of empirical networks: namely, the neural network of the nematode *C. elegans* [1], [34], the power grid of the western United States [1], the protein-protein interaction network of the yeast *S. cerevisiae* [35], a dolphin social network [36], a high-energy physics collaboration network [37], and a network science collaboration network [38]. The basic properties of these networks are summarized in Table 2. We note that the neural network of *C. elegans* is naturally a directed network [34]. Here, however, following a common practice in network studies, we shall ignore the orientation of this network (see, for example, [1]), and consider it as an undirected network.

**Table 2:** The empirical networks we study and their basic properties.

	*N*	*M*	*k*	*κ*	ℓ	*C*	*r*
Social network of frequent associations between dolphins [36]	62	159	5.13	1	3.357	0.258	−0.043
Coauthorships between scientists posting preprints on the High-Energy TheoryE-Print Archive [37]	8361	15751	3.77	1332	7.025	0.442	0.294
Coauthorship network of scientists working on network theory and experiment [38]	1589	2742	3.45	396	5.823	0.637	0.462
Network representing the topology of the Western States Power Grid of the United States [1]	4941	6594	2.67	1	18.989	0.080	0.003
Neural network of the worm *C. elegans* [1], [34]	297	2148	14.465	1	2.455	0.292	−0.163
Network of protein-protein interactions in the yeast *S. cerevisiae* [35]	2361	7182	6.084	101	4.376	0.130	−0.085

Number of vertices 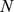, number of edges 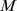, average degree 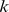, number of connected components 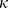, average path length 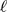, clustering coefficient 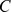, and coefficient of assortativity 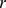.

**Figure 7:**
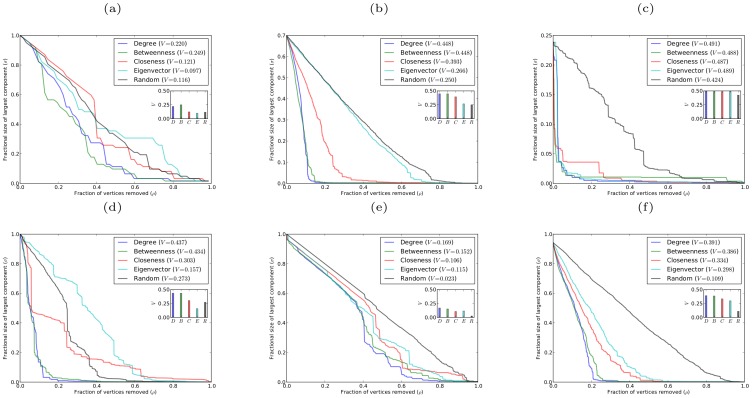
Robustness against simultaneous targeted attack for some empirical networks. (a) dolphin social network; (b) high-energy physics collaboration network; (c) network science collaboration network; (d) power grid network; (e) neural network of *C. elegans*; (f) protein-protein interaction network of *S. cerevisiae.*

Figure 7 shows the robustness results for these empirical networks. These results are broadly consistent with those found for the different classes of model networks. The clearest feature of the graphs and the corresponding 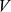-indices is that in most cases simultaneous targeted attack by degree and betweenness are the most effective means of degrading the networks. In all cases these two centrality measures are of very similar efficacy, with degree being slightly better in some cases and betweenness in others. It is interesting to note that the network science collaboration network, which has a clustering coefficient of 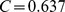, exhibits the fragility to simultaneous targeted attack that was observed in model networks with high clustering coefficient. We believe that the lack of robustness exhibited by this network is an empirical example of the previously conjectured general property of networks exhibiting decreased robustness as their clustering coefficient increases. We note that this network also has a high assortativity coefficient of 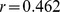, however, we do not believe this to be the cause of the fragility of the network since we found in our studies of model networks that robustness increases as the assortativity coefficient increases.

### Sequential Targeted Attack

We next turn to the study of the robustness of networks under sequential targeted attack.

**Figure 8:**
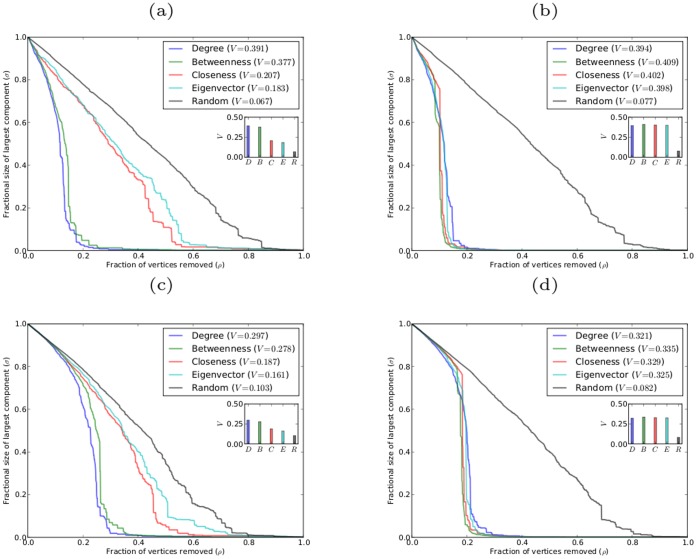
Robustness of model networks with power-law and exponential degree distributions, with 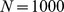, and 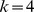. (a)(b) scale-free network against simultaneous and sequential attacks, respectively; (c)(d) exponential network against simultaneous and sequential attacks, respectively.

Figure 8 shows the robustness results for sequential targeted attack on networks with power-law and exponential degree distributions (in comparison with the corresponding results for simultaneous targeted attack). It is immediately apparent from the graphs of 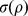 and the corresponding 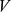-indices that networks are degraded quite differently under sequential targeted attack as compared to simultaneous targeted attack. First, it is clear that networks exhibit greater vulnerability to sequential attack based on any centrality measure than is the case under simultaneous attack. Second, the large difference in the efficacy of targeted attack according to different centrality measures (e.g., targeting according to degree as opposed to targeting by eigenvector centrality) that occurs with simultaneous attack is no longer present with sequential attack. With sequential targeted attack the most effective means of degrading these networks is through removing vertices in decreasing order of betweenness centrality. Next most effective are closeness and eigenvector centrality, and degree centrality is the least effective. This is in stark contrast to the situation for simultaneous targeted attack in which removing vertices in decreasing order of degree proved to be consistently superior to any other centrality measure. However, there are only small differences in the effectiveness of sequential targeted attack based on different centrality measures.

**Figure 9:**
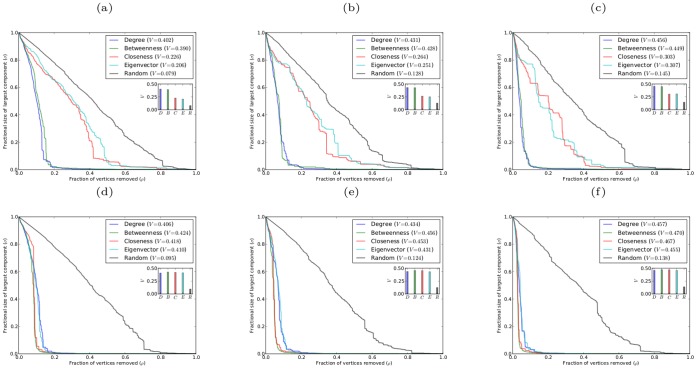
Robustness of model scale-free networks with 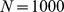, 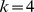, and different values of the clustering coefficient 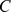. (a)(b)(c) networks with 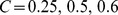, respectively, against simultaneous attack; (d)(e)(f) networks with 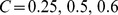, respectively, against sequential attack.

**Figure 10:**
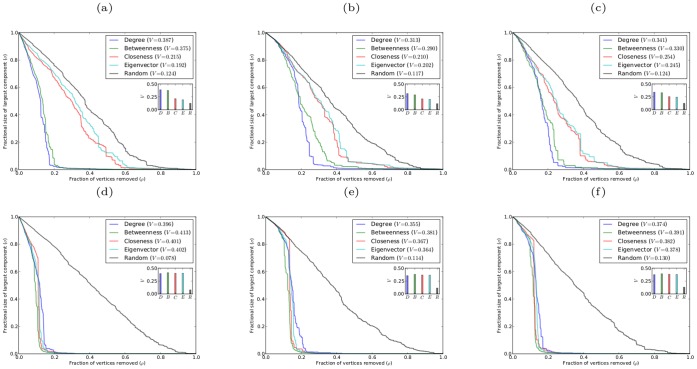
Robustness of model scale-free networks with 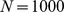, 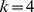, and different values of the coefficient of assortativity 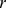. (a)(b)(c) networks with 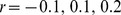, respectively, against simultaneous attack; (d)(e)(f) networks with 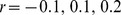, respectively, against sequential attack.

Figure 9 and Figure 10 show the robustness results for scale-free networks with different clustering coefficients and with different degrees of assortativity, respectively. The results for both classes of networks follow the same pattern as found for networks without clustering or assortativity. In all cases for sequential attack the networks are most effectively degraded by removing vertices in decreasing order of betweenness centrality, while removing vertices in reverse order of degree is the least effective method. Again there are only small differences in the effectiveness of sequential targeted attack based on different centrality measures; and in the case of networks with clustering, attack based on betweenness and closeness are almost indistinguishable in efficacy, as are attack based on eigenvector and degree centrality.

**Figure 11:**
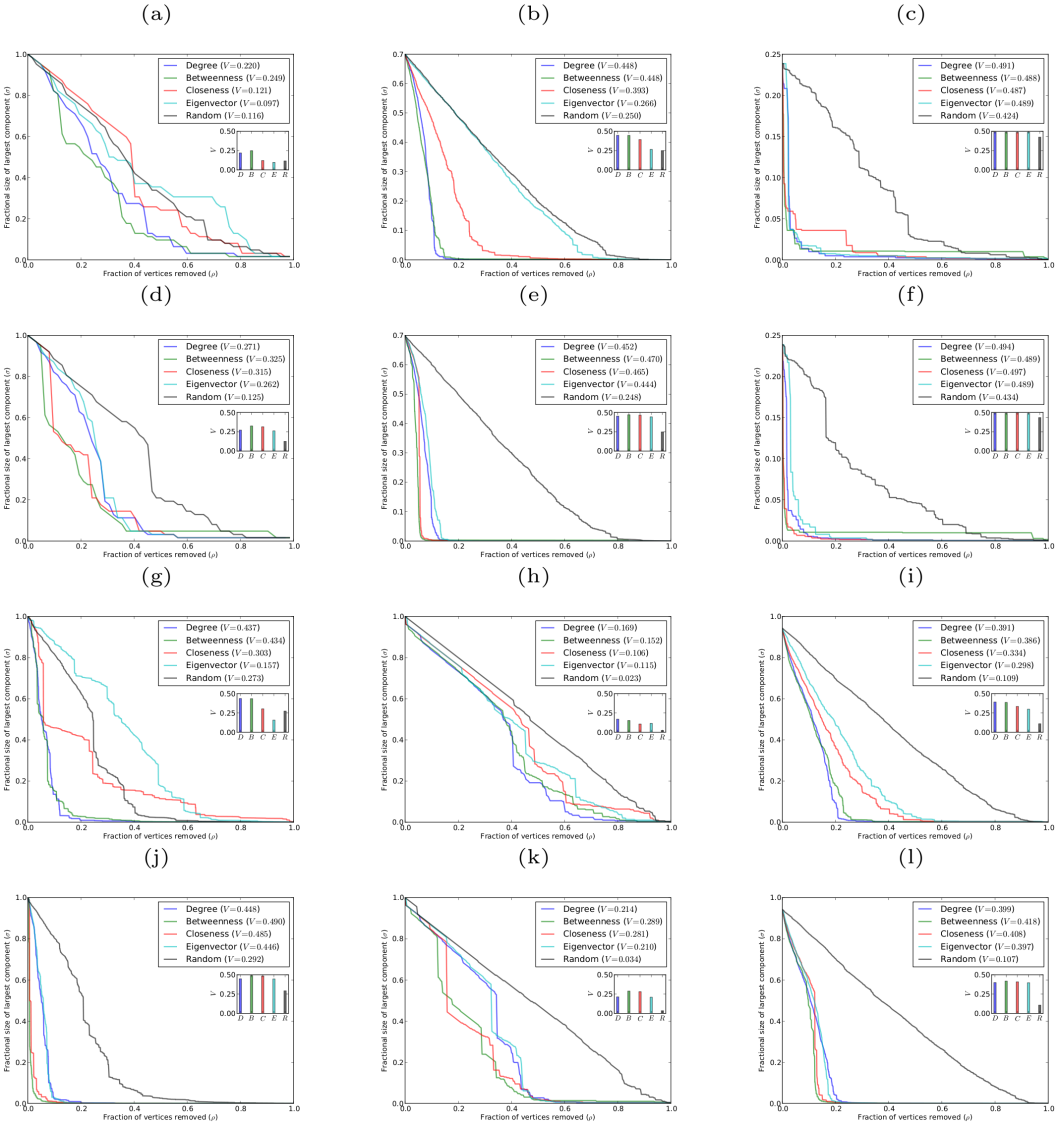
Robustness of empirical networks. (a) dolphin social network against simultaneous attack; (b) high-energy physics collaboration network against simultaneous attack; (c) network science collaboration network against simultaneous attack; (d) dolphin social network against sequential attack; (e) high-energy physics collaboration network against sequential attack; (f) network science collaboration network against sequential attack; (g) power grid network against simultaneous attack; (h) neural network of *C. elegans* against simultaneous attack; (i) protein-protein interaction network of *S. cerevisiae* against simultaneous attack; (j) power grid network against sequential attack; (k) neural network of *C. elegans* against sequential attack; and (l) protein-protein interaction network of *S. cerevisiae* against sequential attack.

We have also studied the robustness under sequential targeted attack of the six empirical networks. Figure 11 shows these results. The pattern that emerges here is that sequentially targeting vertices according to betweenness centrality is the most effective means of degrading these networks. Sequentially targeting vertices by closeness centrality is almost as effective as using betweenness centrality. It is interesting to note that the lack of any great difference in the effectiveness of sequentially targeting vertices according to different centrality measures that was observed for model networks does not hold in general for these empirical networks. Both the neural network and the dolphin network show significant differences in the efficacy of sequential targeting based on different centrality measures. In both cases, sequentially targeting according to betweenness and closeness are most effective, while targeting based on eigenvector and degree centrality are considerably less so.

The V-indices under both simultaneous and sequential targeted attack for all the synthetic and empirical networks discussed here are summarized in Table 3.

**Table 3:** Comparison of the 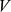-indices of model and empirical networks in the case of simultaneous and sequential attacks.

	Degree	Betweenness	Closeness	Eigenvector	Random
Power-law network ( 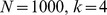 )	0.391	0.377	0.207	0.183	0.067
	0.394	0.409	0.402	0.398	0.077
Exponential network ( 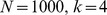 )	0.297	0.278	0.187	0.161	0.103
	0.321	0.335	0.329	0.325	0.082
Scale-free network with clustering ( 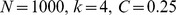 )	0.402	0.390	0.226	0.206	0.079
	0.406	0.424	0.418	0.410	0.095
Scale-free network with clustering ( 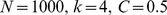 )	0.431	0.428	0.264	0.251	0.128
	0.434	0.456	0.453	0.431	0.124
Scale-free network with clustering ( 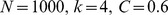 )	0.456	0.449	0.303	0.307	0.145
	0.457	0.470	0.467	0.455	0.138
Scale-free network with assortativity ( 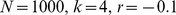 )	0.387	0.375	0.215	0.192	0.124
	0.396	0.413	0.401	0.402	0.078
Scale-free network with assortativity ( 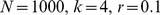 )	0.313	0.290	0.210	0.202	0.117
	0.355	0.381	0.367	0.364	0.114
Scale-free network with assortativity ( 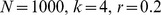 )	0.341	0.330	0.254	0.245	0.124
	0.374	0.391	0.382	0.378	0.130
Dolphin social network	0.220	0.249	0.121	0.097	0.116
	0.271	0.325	0.315	0.262	0.125
High-energy physics collaboration network	0.448	0.448	0.393	0.266	0.250
	0.452	0.470	0.465	0.444	0.248
Network science collaboration network	0.491	0.488	0.487	0.475	0.443
	0.494	0.489	0.497	0.489	0.434
Power grid network	0.437	0.434	0.303	0.157	0.273
	0.448	0.490	0.485	0.446	0.292
Neural network of *C. elegans*	0.169	0.152	0.106	0.115	0.023
	0.214	0.289	0.281	0.210	0.034
Protein-protein interaction network of *S. cerevisiae*	0.391	0.386	0.334	0.298	0.109
	0.399	0.418	0.408	0.397	0.107

Comparison of the 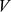-indices of model and empirical networks in the case of simultaneous (top row) and sequential (bottom row) attacks by degree, betweenness, closeness, and eigenvector centralities.

**Figure 12:**
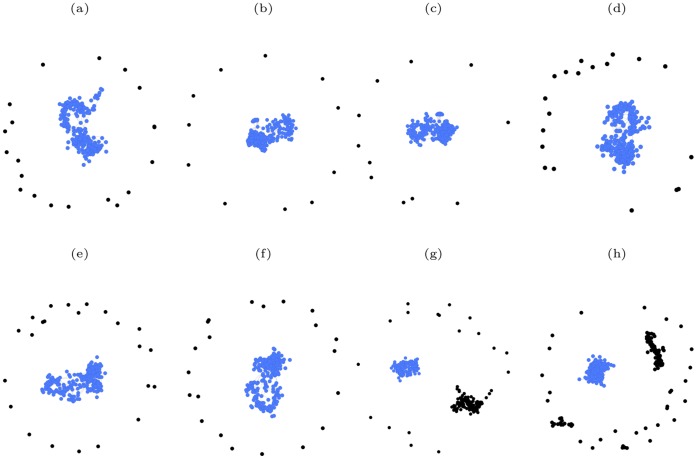
A comparison of the structure of the neural network of *C. elegans* when 20% of the vertices have been removed according to both simultaneous and sequential attack, in decreasing order of degree, eigenvector, closeness and betweenness centrality measures. (a)(b)(c)(d) simultaneous attack based on degree, eigenvector, closeness and betweenness centrality measures, respectively; (e)(f)(g)(h) sequential attack based on degree, eigenvector, closeness and betweenness centrality measures, respectively. For clarity, the vertices in the largest component are colored blue, while all other vertices are colored black. The relative size of the largest component, 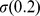, for the different cases are: (a) 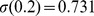; (b) 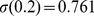; (c) 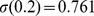; (d) 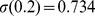; (e) 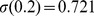; (f) 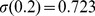; (g) 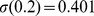; (h) 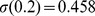.

**Figure 13:**
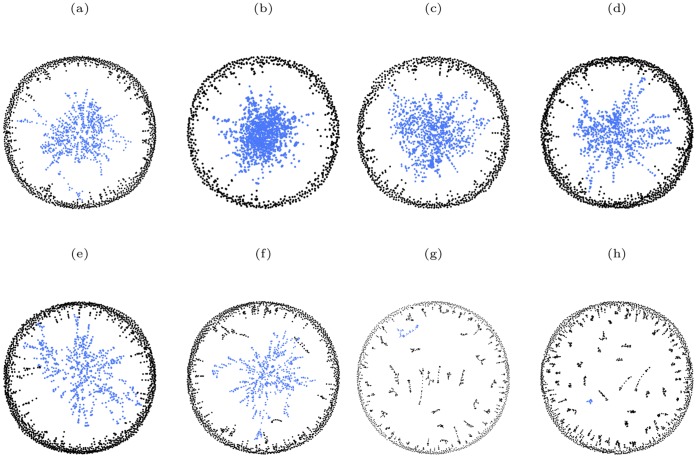
A comparison of the structure of the protein-protein interaction network of *S. cerevisiae* when 15% of the vertices have been removed according to both simultaneous and sequential attack, in decreasing order of degree, eigenvector, closeness and betweenness centrality measures. (a)(b)(c)(d) simultaneous attack based on degree, eigenvector, closeness and betweenness centrality measures, respectively; (e)(f)(g)(h) sequential attack based on degree, eigenvector, closeness and betweenness centrality measures, respectively. For clarity, the vertices in the largest component are colored blue, while all other vertices are colored black. The relative size of the largest component, 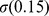, for the different cases are: (a) 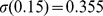; (b) 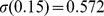; (c) 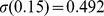; (d) 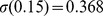; (e) 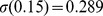; (f) 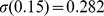; (g) 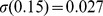; (h) 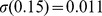.

Finally, it is interesting to visualize the structure of various networks under both simultaneous and sequential targeted attack according to different centrality measures. As an illustration of such structure we show in Figure 12 the neural network of *C. elegans* when a fraction of the vertices have been removed through both simultaneous and sequential targeted attack according to the four centrality measures. The corresponding results for the protein-protein interaction network of *S. cerevisiae* are shown in Figure 13. In both cases it is clear that the sizes of the largest components do not change greatly for simultaneous targeted attack based on any of the four centrality measures. In contrast to this, the size of the largest component for both networks is significantly smaller for sequential targeted attack according to betweenness and closeness than for sequential targeted attack according to eigenvector and degree centrality.

## Discussion

Complex networked systems occur in many areas of the natural and social sciences, and also in many technological areas. In view of the prevalence of such systems, it is of great importance to understand how the failure of their component parts impacts the integrity of the overall system. This issue is closely related to understanding how the structure of a complex network changes as the vertices in it are removed. Here we have investigated how the structure of complex networks changes as vertices are removed according to simultaneous and sequential targeted attack based on degree, betweenness, closeness, and eigenvector centrality measures. Our results extend those previously found in [14] for targeted attack based only on degree and betweenness. For simultaneous attack against most classes of model networks it is the case that the most vulnerable vertices are those with highest degree. Thus, removing vertices in decreasing order of degree is most effective at degrading these types of networks. It is rather striking that degree, which is a purely local centrality measure, is more effective at identifying those vertices whose removal most significantly impact the structure of the network than the other three centrality measures, which are more complex and non-local in nature. A significant caveat concerning this result is that for assortative networks removing vertices in decreasing order of betweenness centrality is initially more effective at degrading such networks. Once a sufficient fraction of the vertices have been removed according to betweenness it again becomes more effective to remove vertices in reverse order of degree.

For sequential targeted attack, the results are significantly different. For sequential attack against all of the networks we have considered removing vertices in reverse order of betweenness is the most effective means of degrading the network structure. Removing vertices in decreasing order of closeness is in all cases almost as effective as removing them based on betweenness. Eigenvector and degree centrality are the least effective methods of exposing network vulnerability under sequential attack. It is important to note, however, that for all of the classes of model networks that we have considered the differences in the effectiveness of sequential targeted attack based on any of the four centrality measures is small. In contrast to this is the interesting fact that for empirical networks there can be significant differences in the effectiveness of sequential targeted attack based on different centrality measures. In these cases, betweenness and closeness prove to be the most (and almost equally) effective means of targeting vertices for removal, while eigenvector and degree centrality are the least effective. It appears that this difference in the vulnerability of model and empirical networks to sequential targeted attack based on various centrality measures reflects subtle structural properties that are possessed by certain empirical networks but are absent from model networks, even when the model networks have similar degree distributions, clustering coefficients and coefficients of assortativity to the empirical networks under consideration. Elucidation of the nature of these structural differences appears to be an interesting and important avenue for future research.
